# Plasma miR-200b in ovarian carcinoma patients: distinct pattern of pre/post-treatment variation compared to CA-125 and potential for prediction of progression-free survival

**DOI:** 10.18632/oncotarget.5766

**Published:** 2015-09-21

**Authors:** Nikiforos-Ioannis Kapetanakis, Catherine Uzan, Anne-Sophie Jimenez-Pailhes, Sébastien Gouy, Enrica Bentivegna, Philippe Morice, Olivier Caron, Claire Gourzones-Dmitriev, Gwénaël Le Teuff, Pierre Busson

**Affiliations:** ^1^ UMR8126 CNRS, Université Paris-Sud, Université Paris-Saclay, Gustave Roussy, F-94805, Villejuif, France; ^2^ Department of Surgery, Gustave Roussy, F-94805, Villejuif France; ^3^ Department of Oncological Medicine, Gustave Roussy, F-94805, Villejuif France; ^4^ Department of Biostatistics and Epidemiology, Gustave Roussy, F-94805, Villejuif France; ^5^ U1018 INSERM, CESP, Université Paris-Sud, Université Paris-Saclay, F-94085, Villejuif, France

**Keywords:** ovarian cancer, plasma, microRNA, miR-200b, progression-free survival

## Abstract

Ovarian carcinomas (OvCa) are highly heterogeneous malignancies. We investigated four circulating plasma microRNAs (miR-21, miR-34a, miR-200b and miR-205) as candidate biomarkers. Using qPCR, we assessed the plasma concentration of these markers in 101 women, including 51 previously untreated OvCa patients, 25 healthy women and 25 patients bearing benign pelvic lesions. For a subset of 33 OvCa patients, the assay was repeated at the end of the primary treatment. The pattern of variations (post- minus pre-treatment) of concentration was compared to that of CA-125. A Cox regression model was used to study the association between variations and the progression-free survival (PFS). Plasma miR-200b proved to have a greater average concentration in OvCa samples (median 2^−ΔΔCt^ = 15.18) than in samples linked to non-malignant lesions (median 2^−ΔΔCt^ = 1.26, *p*-value = 0.0004). Its concentration was highly heterogeneous among OvCa patients, without any correlations with the FIGO stage and the pre-treatment CA-125 level. The decrease in CA-125 concentration was constant and often dramatic, while the variations of miR-200b concentration were much more diverse. The variation of miR-200b was marginally associated with the PFS (hazard ratio=2.95 95%CI=[0.94; 9.28], *p*=0.06) while miR-200b as a continuous time-dependent variable was significantly associated (HR=1.06 [1.02; 1.10], *p*=0.003). This study is the first direct empirical evidence that miR-200b can provide additional information, independent of CA-125 in OvCa patients.

## INTRODUCTION

In developed countries, ovarian carcinoma (OvCa) is the most lethal gynecologic cancer. High-grade serous ovarian carcinoma (HGSOC) is the most frequent (60%) and aggressive type of ovarian malignancies [1, 2]. Its bad prognosis is the result of late-stage discovery and unpredictable, as well as heterogeneous, response to treatment. At present, conventional imaging remains insufficient for the management of ovarian tumors, lacking sensitivity for the detection of small tumors and minimal residual disease. That is why the evaluation of treatment response often requires a surgical approach - second-look laparotomy or laparoscopy - to allow direct observation of the lesions.

Most ovarian carcinomas are found in stage III (extension to the peritoneum and/or lymph nodes) or IV, with a five-year survival rate inferior to 30% [1]. Surgical cytoreduction (debulking) is a key component of any curative therapeutic strategy. Standard chemotherapy protocols combine a platinum salt and a taxane. Between 10% and 20% of ovarian tumors are initially resistant to this combination. The addition of Avastin to the platinum-taxane combination improves the tumor response in some cases [3]. The need for new biomarkers for OvCa is acute not only for early detection but also for the assessment of prognosis and response to treatment. CA-125 and other protein biomarkers have limited prognostic value [4].

MicroRNAs are small, single-stranded non-coding RNAs, about 19-25 nt long, which play key roles in the regulation of gene expression at the post-transcriptional level [5]. Multiple alterations of tumor microRNAs have been reported in most ovarian malignancies [6]. They are often released in the extracellular medium by healthy and malignant cells, associated to various carriers [7-9]. They are protected from RNases and can diffuse from the tumor interstitial fluid to the blood stream [10]. Several investigators, starting from 2008, published data on circulating microRNAs in the context of ovarian carcinomas [9, 11, 12]. However, despite a number of subsequent publications, so far no microRNA or set of microRNAs has been introduced in the clinics as biomarkers for ovarian carcinomas.

Our initial aim was the reproduction of previous findings on 4 circulating miRNAs in 51 women hospitalized for primary ovarian cancer. High concentrations in plasma samples from OvCa were found only for miR-200b. In contrast, the distribution of the 3 other selected miRNAs was highly similar in samples from untreated patients bearing OvCa or benign tumors. A longitudinal study (pre/post-treatment) was conducted, assessing the plasma levels of miR-200b. Our findings show that circulating miR-200b can provide additional information to that provided by CA-125. In contrast to CA-125, variations of miR-200b (post- minus pre-treatment) are marginally significantly associated with progression-free survival (PFS).

## RESULTS

### Patients' characteristics

From 72 OvCa patients initially included in this study, 21 were excluded for various reasons. In consequence, 51 OvCa patients were included with a median follow-up of 39.7 months (range: 1.8; 56.6). The mean age was 62 years (min: 32; max: 81) with 7.8% FIGO stage I (*N* = 4), 3.9% stage II (*N* = 2), 82.4% stage III (*N* = 42) and 5.9% stage IV (*N* = 3). The 2 control groups were 25 HW and 25 BT (Supplemental Data, Figure 1). According to the modalities of the primary treatment, the 33 sequentially studied OvCa patients were classified in 3 categories, as seen in Table 1 (thereafter designated as “primary treatment categories”). Category 1 represents tumors which remained non-resectable at all stages of the follow-up and were treated exclusively with chemotherapy. Category 2 included tumors which were initially non-resectable and firstly treated with neo-adjuvant chemotherapy for tumor reduction, followed by debulking surgery and adjuvant chemotherapy. In category 3, the patients' tumors allowed a direct debulking surgery completed by adjuvant chemotherapy.

**Figure 1:**
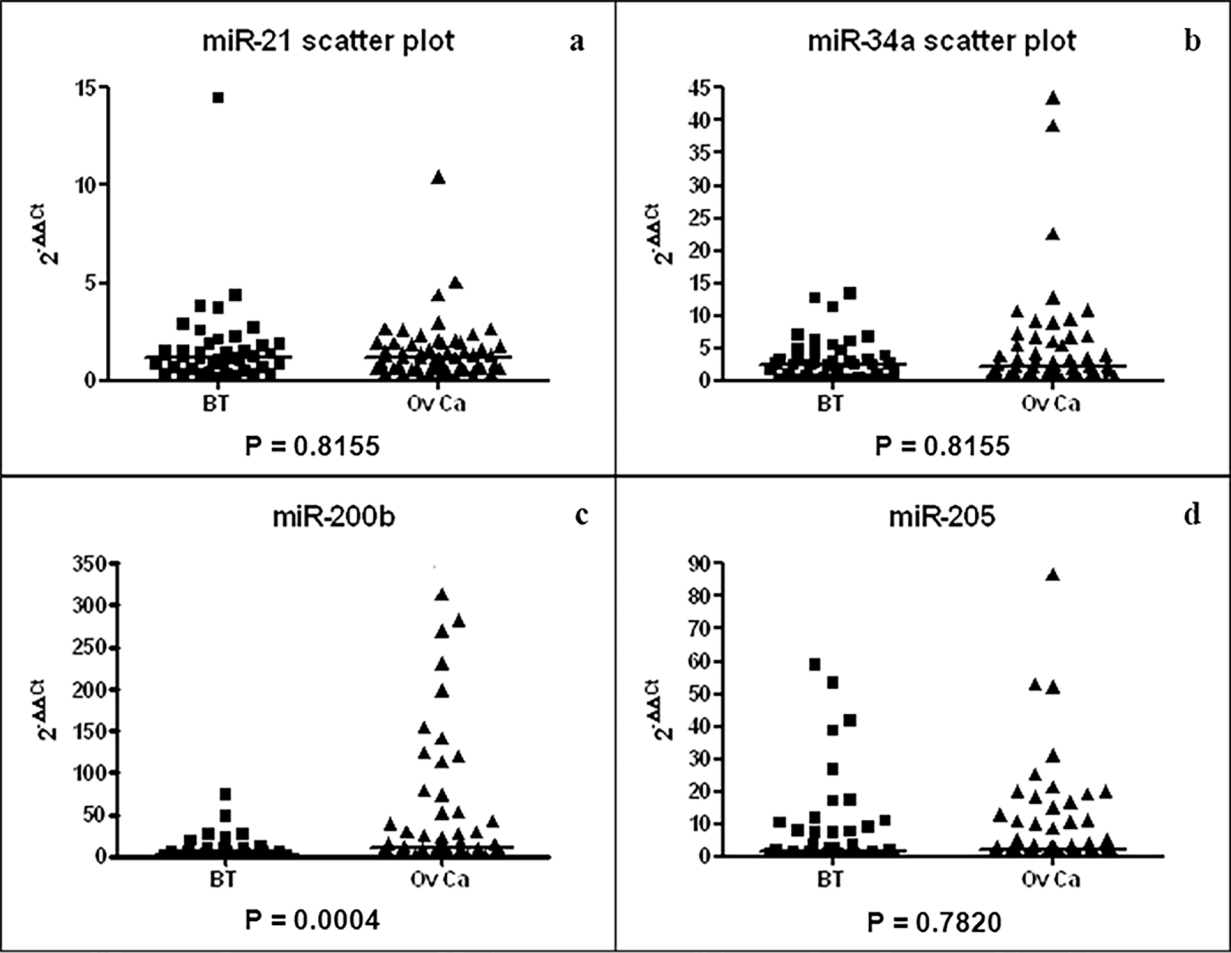
Scatter plots of miR-21 (a), miR-34a (b), miR-200b (c) and miR-205 (d) plasma concentrations for BT (N = 25) and OvCa (N = 51) patients Median 2^−ΔΔCt^ (horizontal lines) is 1.17 (0.62; 1.85) (BT) and 1.18 (0.64; 1.91) (OvCa) for miR-21, 2.50 (0.83; 4.57) (BT) and 2.33 (1.01; 6.41) (OvCa) for miR-34a and 1.76 (0.34; 8.84) (BT) and 3.01 (0.86; 12.46) (OvCa) for miR-205.

**Table 1:** Variations (post- minus pre-treatment) of the plasma concentrations of CA-125 and miR-200b (median [Q1; Q3]) by primary treatment category and overall (*N*=33)

	Primary treatment categories*	Patients	Pre-treatment	Post-treatment	Pre/post-treatment variation (Δ)
CA-125	1) Unresectable tumor	9	2819.0 [1264.0; 8455.0]	31.6 [20.0; 57.0]	−2778.0 [−6855.0; −1254.0]
	2) Debulking after chemo	14	573.3 [235.0; 2858.0]	10.5 [7.4; 26.0]	−557.3 [−2645.0; −227.0]
	3) Direct debulking	10	200.5 [39.0; 230.0]	11.0 [7.0; 16.0]	−187.5 [−225.0; −27.0]
	Total	33	521.5 [184.0; 2819.0]	14.0 [8.0; 31.6]	−499.6 [−2645.0; −166.0]
miR-200b	1) Unresectable tumor	9	52.4 [14.4; 165.7]	136.8 [32.4; 239.6]	+7.7 [−1.0; 78.6]
	2) Debulking after chemo	14	16.1 [4.9; 53.1]	7.0 [1.3; 67.7]	−4.2 [−25.8; 5.1]
	3) Direct debulking	10	69.3 [17.5; 202.7]	22.7 [13.2; 54.6]	−20.9 [−157.4; 34.7]
	Total	33	30.3 [11.8; 93.4]	24.9 [4.4; 104.9]	0 [−49.4;34.7]

* 1: Non-resectable tumor at all stages of the treatment treated exclusively by chemotherapy; 2: Initially non-resectable tumor, treated by neo-adjuvant chemotherapy for tumor reduction, debulking surgery and adjuvant chemotherapy; 3: Initial debulking surgery followed by adjuvant chemotherapy.

### Circulating miR-200b is significantly more abundant in patients bearing ovarian cancer than benign pelvic lesions

Out of the 4 candidate microRNAs (miR-21, miR-34a, miR-200b and miR-205) suspected to have a frequent high concentration in plasma samples from OvCa patients compared to BT patients, only miR-200b was found with a distinct pattern of distribution in these patients. The concentration median 2^−ΔΔCt^ was 15.18 (Q1: 3.47; Q3: 68.64) in OvCa compared to 1.26 (0.48; 5.73) in BT (*p*-value = 0.0004, Figure 1c). We observed no significant difference of the concentration of miR-21 (*p*-value = 0.82, Figure 1a), miR-34a (*p*-value = 0.82, Figure 1b) and miR-205 (*p*-value = 0.78, Figure 1d) between BT and OvCa groups.

**Table 2:** Prognostic effect of miR-200b and CA-125 in ovarian carcinoma patients according to different statistical approaches First approach: the initial value of marker (*N*=51, 30 progressions); second approach: the variation (ΔmiR-200b and ΔCA-125); third approach: the initial value plus the variation, fourth approach: the marker as a continuous time-dependent covariate.

Models	Univariate	Multivariable^¶^
	HR [95%CI] (*p*-value) ^§^	HR 95%CI (*p*-value) ^§^
**miR-200b**		
Initial value* (*N*=51)	1.00 [0.960;1.042] (0.983)	1.007 [0.962;1.053] (0.775)
^†^Variation (Δ) (*N*=24) Negative Positive	1.00 3.122 [1.130;8.625] (0.028)	1.00 2.326 [0.828;6.535] (0.109)
^†^Initial value* Variation (Δ) Negative Positive	1.007 [0.935;1.085] (0.849) 1.00 3.143 [0.967;10.216] (0.057)	1.05 [0.968;1.139] (0.2397) 1.00 2.953 [0.939;9.281] (0.064)
Time-dependent*^‡^ (*N*=33)	1.063 [1.026;1.101] (<0.001)	1.057 [1.020;1.096] (0.003)
**CA-125**		
Initial value* (*N*=51)	1.00 [0.999; 1.001] (0.518)	1.000 [0.999; 1.001] (0.423)
^†^Variation (Δ) (*N*=24) Negative Positive	NE	NE
Initial value* Variation (Δ) Negative Positive	NE	NE
Time-dependent*^‡^ (*N*=33)	1.001 [0.999; 1.004] (0.198)	1.001 [0.999; 1.003] (0.402)

† landmark analysis at 10 months. 9 patients were excluded. The variation represents the difference of the concentration of one marker between post- and pre-treatment (noted Δ=sample B – sample A)

¶ Multivariable Cox model adjusted on FIGO stage (I, II vs III, IV) and primary treatment categories described in patients' characteristics (1 vs 2, 3)

‡ We used the counting process for time-dependent marker. This allows reclaiming 9 patients excluded when using the landmark analysis

* Hazard ratios reported for 10 units of change in the continuous marker

NE not evaluable because only one patient has an increase of CA-125

§ HR for Hazard Ratios and CI for confidence interval

### Independent distribution of plasma concentrations for miR-200b and CA-125 (*N* = 51)

**Figure 2:**
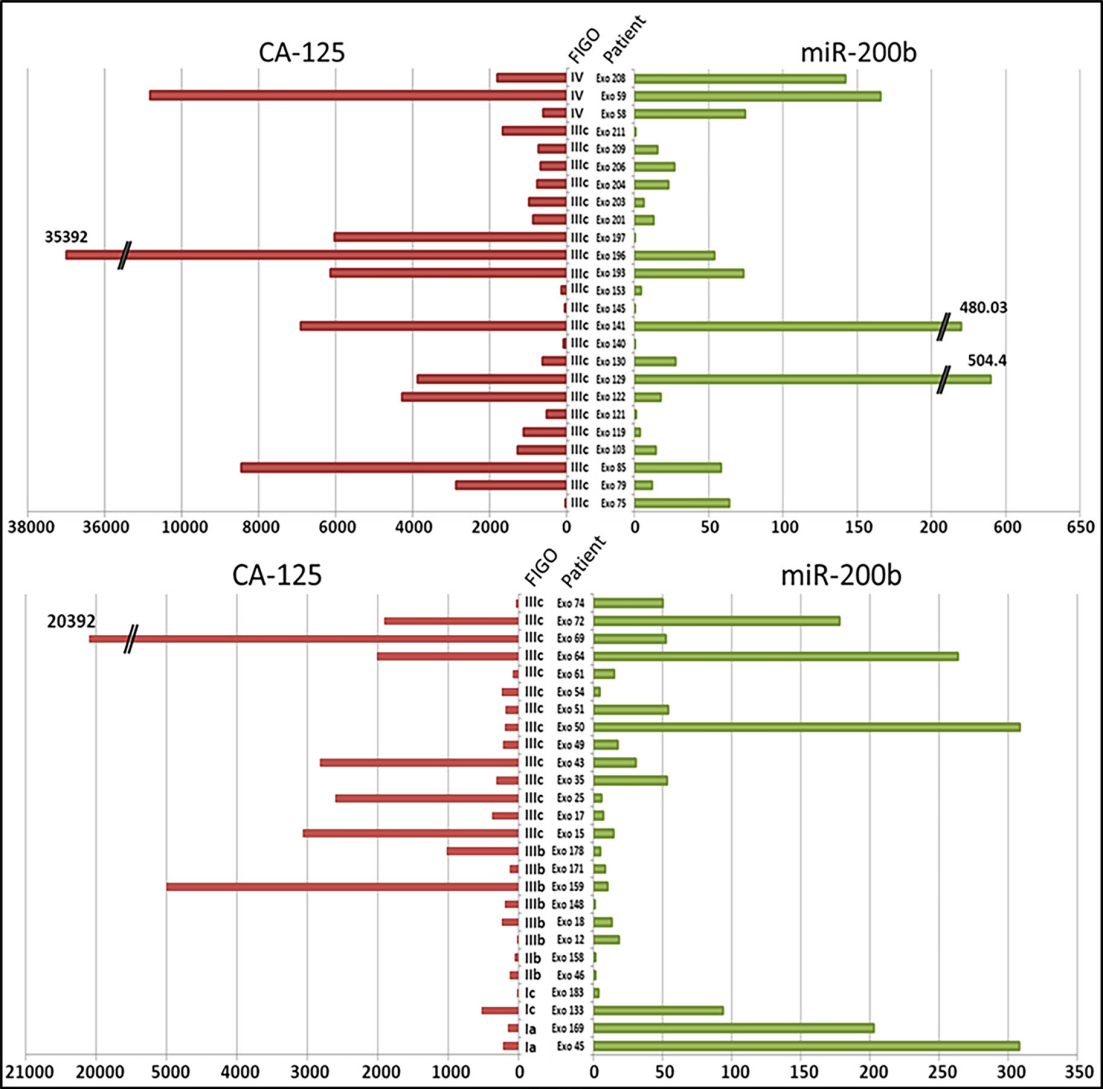
Individual profiles of the pre-treatment concentrations of CA-125 (U/ml) and miR-200b in plasma samples from each OvCa patient (*N* = 51) The concentration of miR-200b was assessed by the 2^−ΔΔCt^ method. The FIGO stages are mentioned between the two histograms, as well as the patients' code.

As shown in Figure 1, the plasma concentrations of miR-200b were highly heterogeneous among OvCa patients. Since we knew that the same was true for CA-125, the standard-of-care biomarker for OvCa, we undertook to make a parallel assessment of CA-125 and miR-200b in the 51 OvCa patients. As shown in Figure 2, we observed no correlation in the distribution of plasma concentrations for CA-125 and miR-200b: many patients had a concentration of CA-125 above the average and a low concentration of miR-200b and vice-versa (R^2^ = 0.0974, Supplemental Figure 3). In addition, there was no significant correlation between both markers and the patients' age (Supplemental Data, Table 3). When compared to the FIGO stage, CA-125 showed a significant correlation with the tumor stage (*p* = 0.01), which was not observed for miR-200b (*p* = 0.93). Regarding the four cases of early disease (stages Ia-Ic), CA-125 was very low in all of them whereas the concentration of miR-200b was above its average concentration for three of them.

### Distinct patterns of variation for CA-125 and miR-200b concentrations prior and after the primary treatment (*N* = 33)

**Figure 3:**
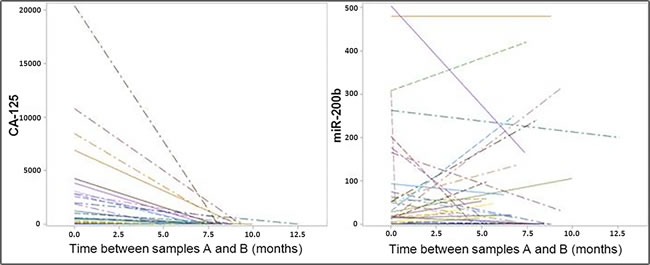
Variations of the plasma concentrations for CA-125 and miR-200b in samples collected prior and after the primary treatment Left: CA-125 exhibits a reduction in its concentration for practically all the cases. Right: Variations in the concentrations of miR-200b are much more diverse; ΔmiR-200b was either negative or positive in about 50% of the cases (16 and 17 patients respectively).

For 33 of the 51 OvCa patients, we were able to collect a second blood sample, at the end of their primary treatment (sample B in addition to the previously mentioned sample A collected prior to any treatment). The variations of CA-125 and miR-200b were designated ΔCA-125 and ΔmiR-200b (concentration of sample B minus concentration of sample A for CA-125 and miR-200b respectively). In almost every case, CA-125 was returning to normal plasma concentrations within the first months of the treatment, even among patients of category 1 (bearing unresectable tumors). At the end of the treatment, CA-125 was either normal (24 patients) or slightly above the limit (5 patients). This is illustrated in Figure 3a by a series of line segments with a negative slope. Consistently, the median CA-125 had the same similar decreasing pattern across the 3 primary treatment categories. The only specific feature of category 1 (unresectable tumors) was a much higher median for the initial concentration of CA-125 (Table 1). When looking at miR-200b, we get a very different picture with generally no variation (median: 0 (Q1: −49.4; Q3: 34.7). There was a remarkable mix of decreasing (16 patients) and increasing (17 patients) variations (Figure 3b). However, the proportion of patients with decreasing concentrations of miR-200b was different depending on the category of tumors: 3/9 (33%) for category 1 (unresectable tumors) versus 13/24 (54%) for categories 2 and 3 (tumors resectable immediately or after neo-adjuvant chemotherapy). Consistently, the median variation was positive for type 1 (median ΔmiR-200b at +7.7) and negative for types 2 and 3 (median ΔmiR-200b at −4.2 and −20.9 respectively) (Table 1).

### Pre/post-treatment variation of plasma miR-200b (ΔmiR-200b) associated with progression-free survival (PFS) (*N* = 33)

**Figure 4:**
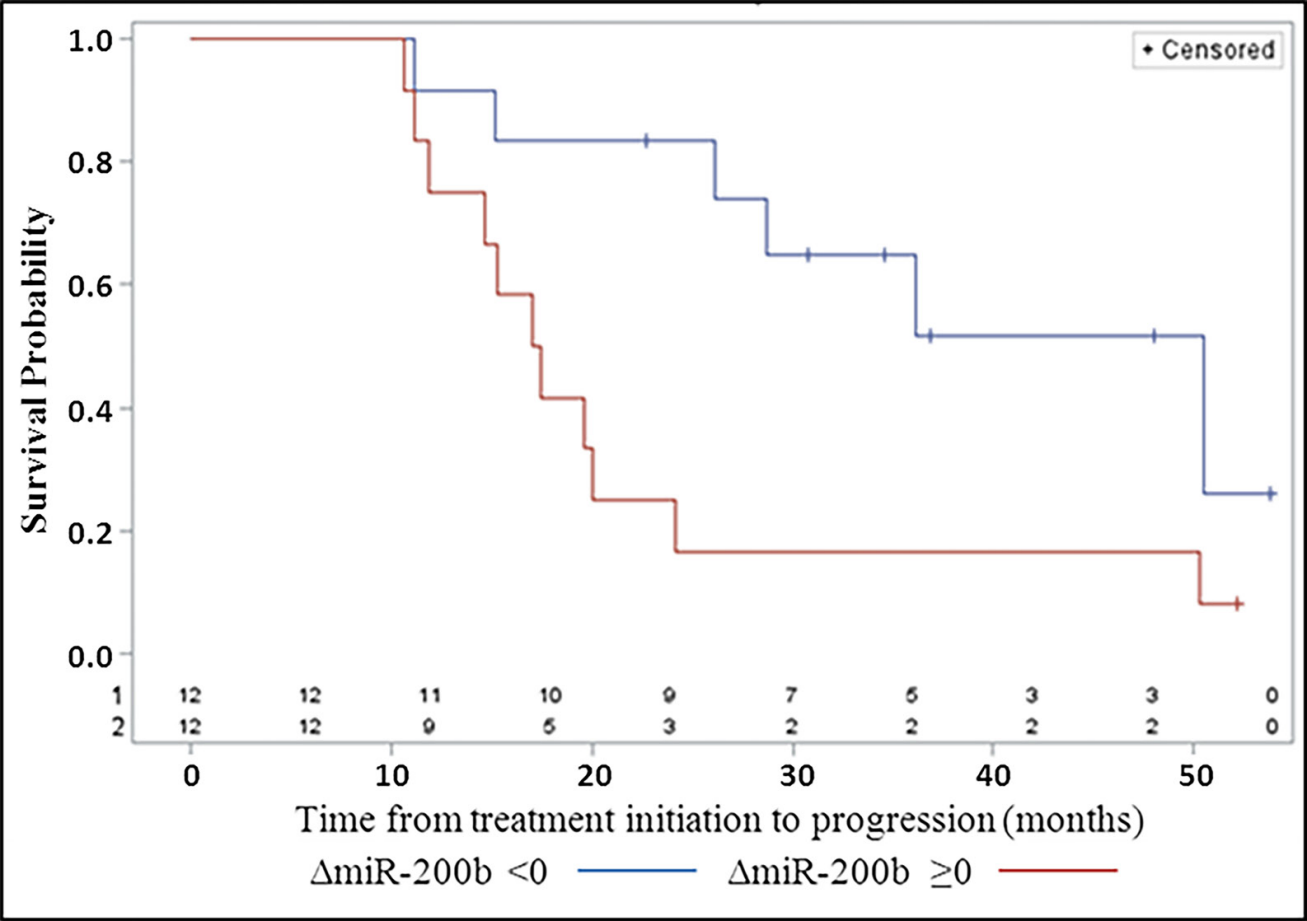
Progression-free survival according to the pre/post-treatment variation ( < 0, ≥0) of the concentration of plasma miR-200b (ΔmiR-200b)

To investigate the association between ΔCA-125 ( < or ≥ 0) and ΔmiR-200b ( < or ≥ 0) with the PFS, a landmark analysis at 10 months was used. This time space represents the highest time interval between evaluation of sample A and sample B. We imputed, for patients without evaluation of sample B at 10 months, the value before this time. Nine patients with an event recorded within the first 10 months had to be excluded from this analysis. Figure 4 shows that among the 24 remaining patients those with a negative ΔmiR-200b had a longer PFS (median: 50.5 months [15.21; NE] than patients with a positive variation (median: 17.3 months [11.2; 24.1] (*p*-value = 0.018).

In order to confirm this observation and to investigate whether the prognostic value of ΔmiR-200b was independent from some major clinical characteristics like tumor extension and the possibility of tumor resection, we used a multivariable analysis based on a Cox model adjusted on tumor stage (I, II vs III, IV) and primary treatment category (1 vs 2, 3). The univariate and multivariable hazard ratios (HR) were 3.12, 95% confidence interval = [1.13; 8.63] (*p* = 0.03), and 2.33, [0.83; 6.54] (*p* = 0.11) to the detriment of ΔmiR-200b≥0. When also adjusting on the initial plasma concentration of miR-200b prior to any treatment as a continuous covariate, univariate and multivariable, HR for the variation were 3.14 [0.97; 10.22] (*p* = 0.06) and 2.95 [0.94; 9.28] (*p* = 0.06), respectively. In other words, the risk of progression was marginally significantly higher in patients with a positive variation of miR-200b compared to patients with a negative variation. It is interesting to note that the initial value of plasma miR-200b was not associated with PFS (*p* = 0.24). The extension of the Cox regression model including miR-200b as a time-dependent covariate (*N* = 33 patients, 25 progressions) showed that the association was now statistically significant (*p* < 0.003 for multivariable analysis). We recorded a 5.7% higher risk of progression for a 10 unit increase of miR-200b concentration. No significant association between CA-125 and PFS was observed when considering the initial value of the marker (*p* = 0.42) or when taking it as a time-dependent covariate (*p* = 0.40).

## DISCUSSION

So far, research on circulating microRNAs in OvCa patients has not resulted in clinical applications, apparently due to a lack of reproducibility [9, 12]. To our knowledge, none of the previous investigators has reported a simultaneous assessment of CA-125, or attempted a sequential assessment of plasma microRNAs to refine the prognosis of OvCa patients.

Our study was focused on 4 microRNAs previously described as often more abundant in the plasma of OvCa patients than in healthy donors or patients bearing benign pelvic tumors [13, 14]. A link with the presence of OvCa was confirmed only for miR-200b. One explanation might be our choice of distinct primers based on LNA technology, which are probably more specific. The distribution of miR-200b concentrations in OvCa plasma samples was highly heterogeneous and not correlated to CA-125 concentrations. Using plasma samples collected from 33 OvCa patients before and after the primary treatment, we made two remarkable observations. Firstly, the patterns of variations from sample A to sample B were much more diverse than for CA-125. Secondly, the sense of variation, increasing or decreasing concentration, was correlated to the PFS.

Markers of this type are eagerly awaited for patients who benefit from tumor resection either immediately or following neo-adjuvant chemotherapy (in contrast to patients with non-resectable tumors expected to have a very short PFS regardless of any biological parameter). If the variation of miR-200b is validated by future studies as a novel biomarker, it will be a tool of major interest for the management of patients on remission assessed by imaging or second-look surgery. Determining the patients at high risk of relapse could lead to the tailoring of more efficient adjuvant therapy approaches. The confirmation of the predictive value of miR-200b will require the investigation of new series of OvCa patients with greater numbers of sequential samples through the primary treatment. This will allow a better assessment of the kinetics of variation for miR-200b.

MiR-200b should not be regarded as a possible substitute of CA-125 but rather as a complementary marker. Two major advantages of CA-125 in the management of OvCa are its contribution to the initial diagnosis and the post-treatment surveillance. On the one hand, in the presence of a pelvic mass, the detection of the CA-125 strengthens the suspicion of ovarian carcinomas prior to laparoscopy or laparotomy [15]. On the other hand, following a complete remission accompanied by CA-125 normalization, its reascension raises the alarm for a relapse which is often confirmed by medical imaging and diagnostic surgery. However, the CA-125 has several limitations; its pattern and kinetics of decrease following the primary treatment are not always correlated to the amplitude and duration of the tumor response [16, 17]. For reasons which are not entirely elucidated, even with a partial tumor response there is often a dramatic decrease in the plasma concentration of CA-125. Because of its poor sensitivity, many patients with normal CA-125 levels after chemotherapy are found to have persistent disease [18, 19]. Therefore CA-125 and miR-200b could co-exist in the management of OvCa patients. CA-125 will be used for the initial diagnosis or for early detection of relapse whereas miR-200b will be used for the prediction of the PFS duration following clinical remission.

The miR-200 family contains five distinct members: miR-141, miR-200a, miR-200b, miR-200c and miR-429 [20]. A number of publications have shown that they are very abundant in ovarian carcinoma cells [21-23], suggesting that miR-200b detected in the plasma of OvCa patients is derived from material released by the malignant cells. However, at least a fraction of this circulating microRNA may derive from cells of the tumor micro-environment. Expression of miR-200b has been reported in proliferative fibroblasts and in a subset of human monocytes [24, 25]. In future studies, it would be necessary to investigate the variation of all the members of the miR-200 family.

It is puzzling to observe that plasma miR-200b can rise in some patients in parallel with a substantial reduction of the tumor mass or even a complete remission. The elucidation of this paradox will be important for its future use as a prognostic marker. At this stage, one can only speculate on the role of hidden tumor cells not easily seen by medical imaging or second-look surgery, or a change of phenotype in cells resistant to the chemotherapy and likely to be involved in tumor relapse.

## MATERIALS AND METHODS

### Biological material

Blood samples were collected prospectively from female patients admitted at Gustave Roussy, with written informed consent under the agreement of the ethical committee (CPP Tarnier-Cochin N°2746, 2010). The blood (10-20 ml) was collected in EDTA tubes and kept at room temperature for less than 2 hours, until plasma separation. Subsequently, it was centrifuged at 1700 g at 20°C for 15 minutes to separate plasma from blood cells. The plasma was homogenized, aliquoted and stored at −80°C, for 6-36 months. As controls, we used 25 samples from healthy women (HW), proved to be free of BRCA1/BRCA2 mutations detected in their family. Besides, we used 2 sets of pathological samples: one obtained from 25 women operated for benign pelvic tumors or lesions (BT hereafter) and a second set obtained prior to any treatment, before a diagnostic coelioscopy, from 72 women treated for malignant ovarian tumors (samples A). Among them, 33 women donated a second sample (samples B) at the end of the primary treatment (treatment including chemotherapy and debulking surgery when feasible), 4-8 months after the initial coelioscopy. For all patients, the follow-up time was at least 6 months from the diagnosis.

### Small RNA extraction

The extraction of all small RNAs was done using the miRCURY RNA isolation kit of Exiqon, optimized for miRNA extraction from biofluids. For each extraction we used 200 μL of undiluted plasma, supplemented with 1 μg of carrier RNA (MS2 phage genomic RNA, Roche) per sample, in order to improve microRNA yield, according to the manufacturer's manual. The total RNA concentration was assessed using a Nanodrop2000 spectrophotometer.

### Reverse transcription to total cDNA

Prior to retrotranscription, the samples were diluted to a final concentration of 5 ng/μl, including the carrier RNA. To exclude immature forms of miRNAs, we used Exiqon's Universal cDNA Synthesis kit II, which adds a poly-A tail to the extracted RNAs before the cDNA synthesis. Two microliters of diluted microRNA extract were mixed with 5 μl of RNase-free water, 2 μl of 5X Reaction buffer and 1 μl of a 10X enzyme mix, containing the reverse transcriptase and a poly-T primer with a 3′ degenerate anchor and a 5′ universal tag. The tubes were incubated at 42°C for 1 hour and at 95°C for 5 minutes, in order to heat-inactivate the enzyme and then stored at −20°C immediately after.

### Selection of microRNA candidates and reference genes

Four microRNAs, hsa-miR-21-5p, hsa-miR-34a-5p, hsa-miR-200b-3p and hsa-miR-205-5p, were selected as candidate biomarkers for OvCa patients on the basis of literature data and MiRandola (the extracellular/circulating microRNAs' online database; http://atlas.dmi.unict.it/mirandola/). Each of them has been reported in at least two publications as exhibiting a high plasma or serum concentration in OvCa patients (Supplemental Data, Table 1). MiR-191 was chosen as an endogenous reference for data normalization (see section of Figure 2 in the Supplemental Data).

### qPCR (quantitative Polymerase Chain Reaction)

The cDNA templates were amplified using Exiqon's miRNA-specific, LNA-enhanced primers (Table 3 in Supplemental Data). After optimization, we fixed the cDNA dilution to 1/40 or 1/20, depending on the specificity of each primer set and the abundance of the target miRNA in the plasma. We used Exiqon's ExiLENT SYBR Green Master Mix, containing the thermostable DNA polymerase, dNTPs and the Mg^2+^ ions needed for DNA replication. We also used ROX, an internal passive fluorescence standard dye, used to correct optical variations. Samples were amplified in triplicates, each well containing 4 μl of the diluted cDNA, 5 μl of SYBR Green Master Mix, 1 μl of the selected primer set and 0.1 μl of ROX dye. The assays were carried on 96-well plates, on a StepOnePlus Real-Time PCR System device, by Applied Biosystems. The amplification protocol was determined according to standard guidelines (Supplemental Data, Table 2) [26].

Samples of all the samples' sets were systematically mixed in the qPCR plates, in order to avoid technical variation. To determine the relative abundance of a given miRNA in a sample, we used the 2^−ΔΔCt^ value [27]. Every Ct for a studied miRNA was normalized with the Ct of the endogenous reference miRNA (ΔCt) and every patient from the BT or OvCa groups was normalized with the average value of the HW group (ΔΔCt value).

### CA-125 quantification

Plasma CA-125 was assayed using the “Access OV-monitor” immunoassay system from Beckman Coulter. The limit of a normal concentration is generally set to 30-35 U/ml.

### Data analysis

Patients' characteristics and treatment modalities were retrieved from Gustave Roussy medical files. The distribution of the concentrations (2^−ΔΔCt^) of the candidate microRNAs was compared between BT and OvCa using the Kruskal-Wallis test. We used the false discovery rate adjustment to control for multiple testing. We studied the correlation between the selected miRNA and CA-125 plasma concentrations through a linear regression and R^2^ after log transformation. The pre/post-treatment variations (noted Δ = sample B - sample A and defined as < or ≥0) of CA-125 and miRNA plasma concentrations were reported (median [Q1, Q3]) by category of primary treatment and overall. Progression-free survival (PFS) was defined as the time from the start of the treatment to the first progression, as defined according to GCIG criteria [28]. Patients without event were censored at the date of the last follow-up examination. The cut-off date was April 30, 2015. We used the Kaplan-Meier method to estimate PFS curves and the log-rank test to compare PFS curves according to status of ΔCA-125 and ΔmiRNA concentrations. We used the Cox regression model to investigate the association between variation (Δ) and PFS and extended this model in considering the biomarkers as a continuous time-dependent covariate. The multivariable analyses were adjusted on stage (I, II versus III, IV) and treatment type (1 vs 2, 3). The Firth's approach was used because of the small sample size. We used the landmark method to assess the prognosis effects of ΔCA-125 and ΔmiRNA changes [29]. *P*-values were two-tailed. We used Graph Pad Prism 4 for the figures and SAS 9.3 for statistical analyses.

## SUPPLEMENTARY MATERIAL FIGURES AND TABLES



## Abbreviations

OvCa: ovarian carcinoma
HGSOC: high-grade serous ovarian carcinoma
BT: benign pelvic lesions or tumors
miRNA: microRNA
PFS: progression-free survival
HW: healthy women
