# Supplementary material for: Plasma miR-200b in ovarian carcinoma patients: distinct pattern of pre/post-treatment variation compared to CA-125 and potential for prediction of progression-free survival
Source: Oncotarget. 2015 Sep 21;6(34):36815–24. doi: 10.18632/oncotarget.5766 (PMC4742212; doi:10.18632/oncotarget.5766)
Supplement: Supplementary file 1 [file oncotarget-06-36815-s001.pdf]

Plasma miR-200b in ovarian carcinoma patients: distinct pattern of pre/post-treatment variation compared to CA-125 and potential for prediction of progression-free survival

Supplementary Material

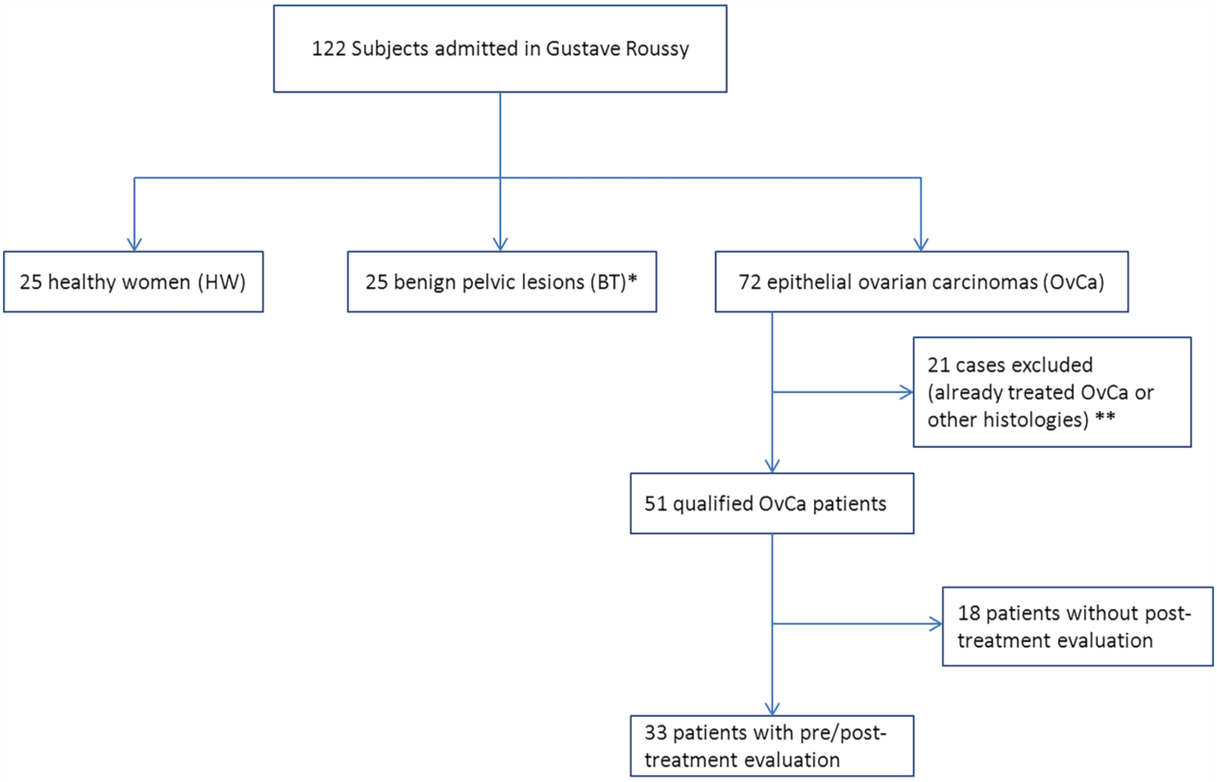

Figure 1: Flow-chart of the subjects included in the study.

*Most cases of BT were ovarian cystadenofibromas, leiomyomas, teratomas, fibrothecomas or various cysts which, after microscopical analysis of the biopsies, were shown to be non-malignant.

**Sixteen cases had received a treatment prior to the first coelioscopy or suffered a cancer within the two last years before the detection of the OvCa. Two patients were excluded, as the tumor was classified as borderline upon histopathological examination. Finally, 3 cases of ovarian tumors were proven to be the result of breast or colon cancer metastases.

Table 1: Candidate miRNAs selected for our experimental procedure.

microRNA	Material	Type of aberration	Reference
miR-21	Plasma, Serum	Increased	[1, 2]
miR-34a	Serum	Increased	[1, 3]
miR-200b	Serum	Increased	[3, 4]
miR-205	Plasma	Increased	[3, 5]

Introduction to Figure 2 - Selection of endogenous reference

Quantitative assessment of microRNAs by real-time PCR requires endogenous references to avoid biases due to RNA extraction and reverse transcription efficiency. Therefore, during preliminary studies, we investigated the potential of 3 miRNAs – hsa-miR-132-3p, hsa-miR-23a-3p and hsa-miR-191-5p – already used for normalization of plasma microRNAs in published studies [1, 6]. Our main selection criteria were their detection at a substantial and consistent level among our 3 groups of plasma samples (HW, BT, OvCa). The consistency of their concentration was evaluated by comparison of the median Ct value using the Kruskal-Wallis test. MiR-132 was rapidly eliminated because of being poorly detected in the majority of the control as well as OvCa subjects (data not shown). MiR-23a was readily detected in most plasma samples but was in fact dependent on the disease state (*p*-value = 0.036). In contrast, the average level of miR-191 was consistent in plasma samples from all 3 categories of donors (median values 31.91, 32.54 and 32.46 for HW, BT and OvCa respectively, *p*-value = 0.13). Therefore, miR-191 was chosen for data normalization (see Figure 2 below).

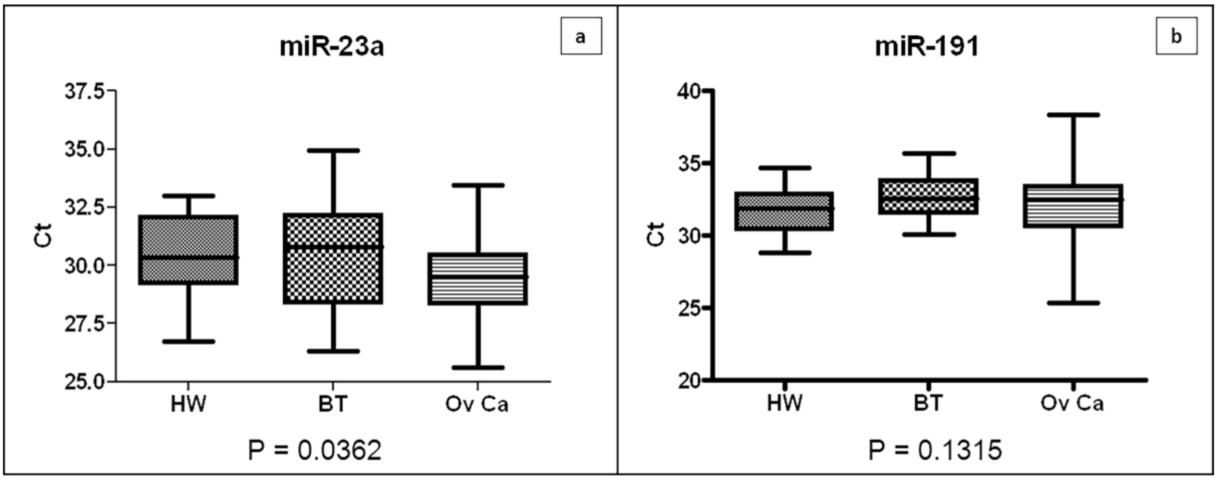

Figure 2: Comparison of miR-23a (a) and miR-191 (b) Ct values in the series of plasma samples from healthy women (HW), women with benign tumors or lesions (BT) and ovarian cancers (OvCa) (N_{HW}=25, N_{BT}=25, N_{OvCa}=51). Ct (cycle threshold) reflects the concentration of targeted miRNAs in each plasma sample (high Ct - low abundance). For miR-23a, the median values for HW, BT and OvCa were significantly different with 30.35, 30.80 and 29.49 respectively. For miR-191, they were not significantly different with 31.91, 32.54 and 32.46 respectively. The reported *p*-values are corrected for multiple testing. The horizontal line in the box interior represents the median.

Table 2: Real-time PCR cycle conditions

**Process step**	**Settings**
Polymerase Activation/Denaturation	95°C, 10 minutes
Amplification	40 cycles at 95°C for 10 seconds and 60°C for 1 minute, with a ramp-rate of 1.6°C/s
Melting Curve	Included

Table 3: Full name, accession number and Exiqon primers' reference number of each of the studied microRNAs.

**microRNA**	**miRBase Accession Number**	**Exiqon Product Number**
hsa-miR-21-5p	MIMAT0000076	204230
hsa-miR-23a-3p	MIMAT0000078	204772
hsa-miR-34a-5p	MIMAT0000255	204486
hsa-miR-132-3p	MIMAT0000426	204129
hsa-miR-191-5p	MIMAT0000440	204306
hsa-miR-200-3p	MIMAT0000318	206071
hsa-miR-205-5p	MIMAT0000266	204487

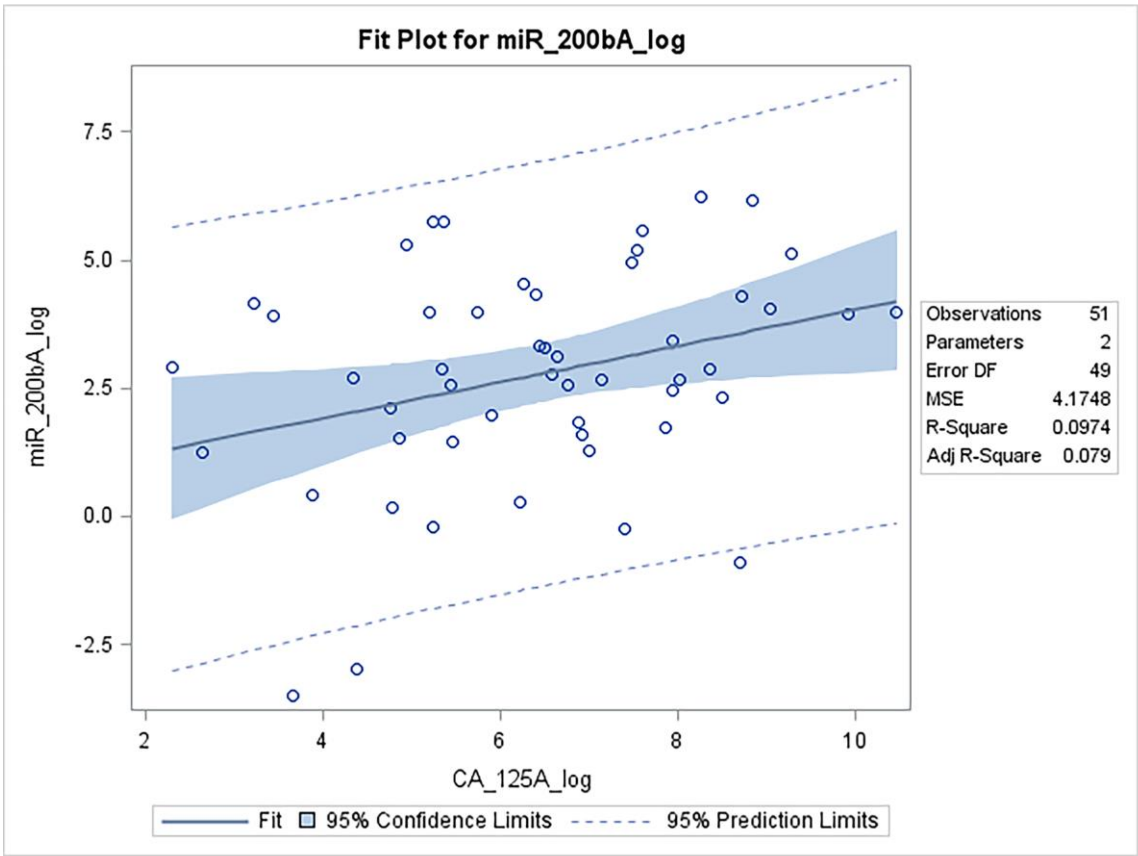

Figure 3: Plot of miR-200b versus CA-125 after log transformation: $R^2 = 0.0974$ (N=51).

Table 4: Correlation between CA-125, miR-200b measured prior to treatment and age, FIGO stage in ovarian cancer patients (*N*=51). Reported *p*-values (Kruskal-Wallis test) are not corrected for multiple testing.

			CA-125<br>Median [Q1; Q3]	miR-200b<br>Median [Q1; Q3]
Age (years)	<65	(*N*=29)	600.0 [190.0; 1652.0]	17.7 [10.0; 53.8]
	≥65	(*N*=22)	1038.0 [119.0; 3058.0]	16.0 [1.5; 73.6]
			p-value= 0.6345	*p*-value= 0.4936
Stage FIGO	I, II	(*N*=6)	130.0 [49.0; 212.0]	48.5 [1.5; 202.7]
	III, IV	(*N*=45)	865.0 [211.0; 2858.0]	17.5 [6.3; 53.8]
			p-value= 0.0119	*p*-value=0.9301

Table 5: Individual fold changes in the concentrations of CA-125 and miR-200b in the samples collected before and after the primary treatment of OvCa patients (*N*=33).

**Patient**	**CA-125 (U/ml)**		**miR-200b ($2^{-\Delta\Delta Ct}$)**	
	**Concentration**	**Fold change**	**Concentration**	**Fold change**
Exo 12*	10→34	x 3.4	18.4→53.1	x 2.9
Exo 15	3058→20	1/153	14.5→6.7	1/2.2
Exo 18	90→5	1/18	13.1→97.8	x 7.5
Exo 25	2600→9.2	1/283	5.6→0.01	1/560
Exo 35	313→6	1/52	53.1→58.2	x 1.1
Exo 43	2819→41	1/69	30.3→250.0	x 8.3
Exo 45	212→11	1/19	308.0→54.6	1/6
Exo 49	211→7	1/30	17.5→24.9	x 1.4
Exo 50	190→16	1/12	308.5→420.3	x 1.4
Exo 51	184→18	1/10	53.8→3.0	1/18
Exo 54	235→8	1/29	4.3→19.5	x 4.5
Exo 58	600→30	1/20	74.6→0.04	1/1865
Exo 59	10816→26	1/416	165.7→32.4	1/5
Exo 64	2009→20	1/101	263.5→199.6	1/1.3
Exo 69	20392→57	1/358	52.4→239.6	x 4.6
Exo 72	1900→7	1/271	177.9→20.6	1/9
Exo 74	31,4→6.7	1/5	50.0→7.3	1/7
Exo 75	25→4	1/6	64.0→14.6	1/4.4
Exo 79	2858→213	1/13	11.8→311.8	x 26.4
Exo 85	8455→1600	1/5	58.2→136.8	x 2.4
Exo 103	1264→10	1/126	14.4→22.0	x 1.5
Exo 121	507→7.4	1/69	1.32→1.32	x 1
Exo 122	4269→40	1/107	17.65→1.31	x 13.5
Exo 129	3861→6	1/644	504.4→166.0	1/3.0
Exo 130	625→3.2	1/195	27.7→104.9	x 3.8
Exo 133	521.5→26	1/20	93.4→67.7	1/1.4
Exo 140	81→8	1/10	0.1→0.3	x 3
Exo 141	6900→41.6	1/166	480.0→480.6	x 1
Exo 145	39→12	1/3	0.03→13.2	x 440
Exo 153	130→31.6	1/4	4.6→48.2	x 10.5
Exo 158	49→14	1/4	1.52→0.51	1/3.0
Exo 169	141→8.2	1/17	202.7→4.4	1/46
Exo 178	1015→110.3	1/9	4,89→2.06	1/2.4

*****The patients are named according to their order of induction in the project.

References

1. Suryawanshi S, Vlad AM, Lin HM et al. Plasma microRNAs as novel biomarkers for endometriosis and endometriosis-associated ovarian cancer. Clin Cancer Res 2013; 19: 1213-1224.
2. Resnick KE, Alder H, Hagan JP et al. The detection of differentially expressed microRNAs from the serum of ovarian cancer patients using a novel real-time PCR platform. Gynecol Oncol 2009; 112: 55-59.
3. Taylor DD, Gercel-Taylor C. MicroRNA signatures of tumor-derived exosomes as diagnostic biomarkers of ovarian cancer. Gynecol Oncol 2008; 110: 13-21.
4. Kan CW, Hahn MA, Gard GB et al. Elevated levels of circulating microRNA-200 family members correlate with serous epithelial ovarian cancer. BMC Cancer 2012; 12: 627.
5. Zheng H, Zhang L, Zhao Y et al. Plasma miRNAs as diagnostic and prognostic biomarkers for ovarian cancer. PLoS One 2013; 8: e77853.
6. Shapira I, Oswald M, Lovecchio J et al. Circulating biomarkers for detection of ovarian cancer and predicting cancer outcomes. Br J Cancer 2014; 110: 976-983.
